# Morphological and molecular characterization of *Eimeria haematopusi* n. sp. (Apicomplexa: Eimeriidae) in an Australian Pied Oystercatcher (*Haematopus longirostris*) (Aves: Charadriiformes)

**DOI:** 10.1007/s11230-024-10152-5

**Published:** 2024-05-13

**Authors:** Jill M. Austen, Belinda Brice, Dandan Liu, Huimin Gao, Bruno P. Berto, Alireza Zahedi, Aileen Elloit, Rongchang Yang

**Affiliations:** 1 School of Agricultural Sciences, Murdoch University, Murdoch, WA 6150 Australia; 2 Kanyana Wildlife Rehabilitation Centre, 120 Gilchrist Road, Lesmurdie, WA 6076 Australia; 3 Jiangsu Co-innovation Centre for Prevention and Control of Important Animal Infectious Diseases and Zoonoses, Yangzhou University, Yangzhou, 225009 Jiangsu People’s Republic of China; 4 Institute of Cash Crops, Hebei Academy of Agriculture and Forestry Sciences, Shijiazhuang, 05005 People’s Republic of China; 5 Departamento de Biologia Animal, Instituto de Ciências Biológicas e da Saúde, Universidade Federal Rural do Rio de Janeiro, BR-465 km 7, Seropédica, RJ 23897-000 Brazil

## Abstract

A novel *Eimeria* Schneider, 1875 species is described from an Australian pied oystercatcher *Haematopus longirostris* Vieillot, in Western Australia. The pied oystercatcher was admitted to the Kanyana Wildlife Rehabilitation Centre (KWRC), Perth, Western Australia in a poor body condition, abrasion to its right hock and signs of partial delamination to its lower beak. Investigation into potential medical causes resulted in a faecal sample being collected and screened for gastrointestinal parasites. Unsporulated coccidian oocysts were initially observed in the faeces and identified as *Eimeria* upon sporulation. The sporulated oocysts (*n* = 20) are ellipsoidal, 20–21 × 12–13 μm in shape and have thick bi-layered walls which are c.2/3 of the total thickness. Micropyle is present, robust and protruding, and occasionally has a rounded polar body attached to the micropyle. Within the oocyst, a residuum, in addition, two to five polar granules are present. There are four ellipsoidal sporocysts 9–11 × 5–6 μm with flattened to half-moon shaped Stieda bodies. Sub-Stieda body and para-Stieda body are absent. The sporocysts contain sporocyst residuums composed of a few spherules scattered among the sporozoites. Within the sporozoites, anterior and posterior refractile bodies are present, but the nucleus is indiscernible. To further characterise the novel *Eimeria* species from *H. longirostris*, molecular analysis was conducted at the 18S ribosomal RNA locus, using PCR amplification and cloning. Two cloned sequences from the novel *Eimeria* were compared with those from other *Eimeria* spp. with the highest genetic similarity of 97.6% and 97.2% from Clone 1 and 2, respectively with *Eimeria reichenowi* (AB544308) from a hooded crane (*Grus monacha* Temminck) in Japan. Both sequences grouped in a clade with the *Eimeria* spp. isolated from wetland birds, which include *Eimeria paludosa* (KJ767187) from a dusky moorhen (*Gallinula tenebrosa* Gould) in Western Australia, *Eimeria reichenowi* (AB544308) and *Eimeria gruis* (AB544336) both from hooded cranes. Based on the morphological and molecular data, this *Eimeria* sp. is a new species of coccidian parasite and is named ***Eimeria haematopusi*** **n. sp**. after its host *H. longirostris*.

## Introduction

The Australian pied oystercatcher (*Haematopus* *longirostris*) is a medium-sized black and white wading bird with distinctive features including a bright orange-red bill, red legs, red eye rings and eyes (Pizzey and Knight, 2007). It is found along much of the Australian and Tasmanian coastline, the south coast of New Guinea and the coasts of the Aru and Kai Islands in Indonesia (Hockey et al., 2020).

The Australian pied oystercatcher belongs to the Order Charadriiformes and the Suborder Charadrii (waders) and is a member of the Haematopodidae family. There are 12 species of oystercatcher in the *Haematopus* genus (Winkler et al., 2020) that include the pied oystercatcher (*H. longirostris *Vieillot), Eurasian oystercatcher (*H. ostralegus *Linnaeus), South Island oystercatcher (*H. finschi *Martens), Chatham oystercatcher (*H. chathamensis *Hartert), variable oystercatcher (*H. unicolor *Forster), sooty oystercatcher (*H. fuliginosus *Gould), American oystercatcher (*H. palliatus* Temminck), African oystercatcher (*H. moquini *Bonaparte), Canarian oystercatcher (*H. meadewaldoi *Bannerman), blackish oystercatcher (*H. ater *Vieillot), magellanic oystercatcher (*H. leucopodus *Garnot) and the black oystercatcher (*H. bachmani *Audubon) (Winkler et al., 2020).

Taxonomical classification places the oystercatchers genetically closest to the ibisbill (Ibidorhynchidae). Together, they form a clade that is most closely related to the avocets and stilts (Recurvirostridae) (Winkler et al., 2020).

*Eimeria* spp. are the eimeriid coccidia with the greatest parasitic diversity, being found in all groups of vertebrates, in addition to invertebrates (Duszynski, 2021). *Isospora* spp. are also widely diverse, but are predominantly found in Passeriformes, but also in other orders such as Strigiformes and Struthioniformes (Berto et al., 2011; 2023; Medina et al., 2019; Woodyard et al., 2019; Coker et al., 2023). Within the Charadriiformes, fewer than 22 species of *Eimeria* (the largest of the Eimeriidae) have been documented to infect members of this order. The *Eimeria* species previously documented include *Eimeria burchinici* (Dzerzhinskii and Kairooaev, 1989) in a stone curlew (*Burhinus oedicencus* Linnaeus), six *Eimeria* species from plovers (Family Charadriidae), five *Eimeria* species from the sandpipers (Family Scolopacidae), *Eimeria stercorariae* Galli-Valerio, 1940 from a parasitic jaeger (*Stercorarius parasiticus* (Linnaeus)), eight *Eimeria* species from gulls and terns (Laridae family) and *Eimeria fraterculae* Leighton and Gajadhar, 1986 from a common puffin (*Fratercula arctica* (Linnaeus)) (Duszynski et al., 2001).

To date, there is only one species of *Eimeria* described from unsporulated oocysts from the *Haematopus* genus, namely *Eimeria haematopi*, isolated from the kidneys of a Eurasian oystercatcher (*H. ostralegus*) (Gottschalk and Prange, 2011). In a later study by Siebert et al. (2012) the detection of coccidian infection in histological kidney sections of two Eurasian oystercatchers (*H. ostralegus*) was evident during the investigation of the health status of seabirds along the North Sea coast of Germany. Unfortunately, no oocysts were isolated or characterized from the study.

In this study, we characterize a new species of *Eimeria* from a wild Australian pied oystercatcher (*H. longirostris*), both morphologically and genetically, and propose the species name *Eimeria haematopusi* **n. sp.**

## Materials and methods

***Sample collection***

An Australian pied oystercatcher (*H. longirostris*) was admitted to the Kanyana Wildlife Rehabilitation Centre (KWRC), Perth, Western Australia, in August 2019 after it had been found on the ground in an unusual location after a crash landing. Initial examination showed that the bird was alert and responsive, but it presented in an extremely poor body condition (1/5). It had reduced grip in both feet and an abrasion to its right hock. The lower beak showed signs of partial delamination. To investigate potential medical causes a faecal sample was collected into a sterile, pre-labelled collection vial so that it could be screened for gastrointestinal parasites. The faecal sample was stored at 4°C until microscopy was performed. Despite intensive treatment, the bird died a few days later.

Ethical review and approval were waived for this study based on the Australian code for the care and use of animals for scientific purposes (2013, 8th Edition and updated in 2021) and Animal Welfare Act 2002 (Western Australia). The faecal samples were collected from the debilitated oystercatcher by volunteers at the KWRC for routine diagnostic purposes with the permission of the Board of the KWRC.

***Morphological observation***

Direct microscopic examination was performed on the faecal sample and a few unsporulated coccidian oocysts were seen. A portion of the faeces was emulsified in a 2% (w/v) potassium dichromate solution (K2Cr2 O7) and stored at 4°C until transport to Murdoch University. Once at Murdoch University, a faecal float was performed on the sample mixture using a saturated sodium chloride and 50% sucrose solution (w/v). A shallow layer (less than 1cm) of the sample mixture was also poured into a Petri dish, placed inside a cupboard, and allowed to sporulate at room temperature (20–22°C). An Olympus DP71 digital micro-imaging camera was used to observe the sporulated oocysts, using the 100× oil immersion objective. Images were captured using a Nomarski contrast imaging system.

***DNA extraction***

Total DNA was extracted from 200 mg of each faecal sample using a Power Soil DNA Kit (MolBio, Carlsbad, California) with some modifications as described by Yang et al. (2015). Briefly, samples were subjected to four cycles of freezing/thawing in liquid nitrogen and boiling water to ensure efficient lysis of oocysts, before being processed using the manufacturer’s protocol.

***Polymerase Chain Reaction (PCR) amplification and cloning of 18S rRNA***

Amplification of the *Eimeria* 18S rRNA region was performed using PCR according to the protocols described by Yang et al. (2016). To identify singular species, cloning of the PCR product was performed as described previously by Yang et al. (2013a) while sequencing and DNA purification were conducted according to previously described protocols (Yang et al., 2013b).

***Phylogenetic analysis***

The two18S rRNA sequences from *E. haematopusi* **n. sp**. were aligned with other closely related *Eimeria* and *Isospora* sequences retrieved from GenBank (Benson et al., 2005) via BLAST (Altschul et al., 1990) searches. Phylogenetic trees of 18S rRNA sequences were constructed using MEGA-X (Kumar et al., 2018) with the most appropriate nucleotide substitution models (TN93 + G + I).

The robustness of nodes within the resulting trees was inferred from 1000 cycles of bootstrap resampling.

***Line drawing***

Line drawings were edited using two software applications from CorelDRAW® (Corel Draw Graphics Suite, Version 2020, Corel Corporation, Canada), i.e., Corel DRAW and Corel PHOTO-PAINT (Yang et al., 2021).

## Results

Low numbers of unsporulated coccidian oocysts were detected by light microscopy and faecal flotation. Upon sporulation, the oocysts were characterised as an *Eimeria* sp. given the presence of 4 sporocysts observed within the oocysts.

**Species description**

Family Eimeriidae Minchin, 1903

Genus *Eimeria* Schneider, 1875

***Eimeria haematopusi*** **n. sp.**

**Fig. 1:**
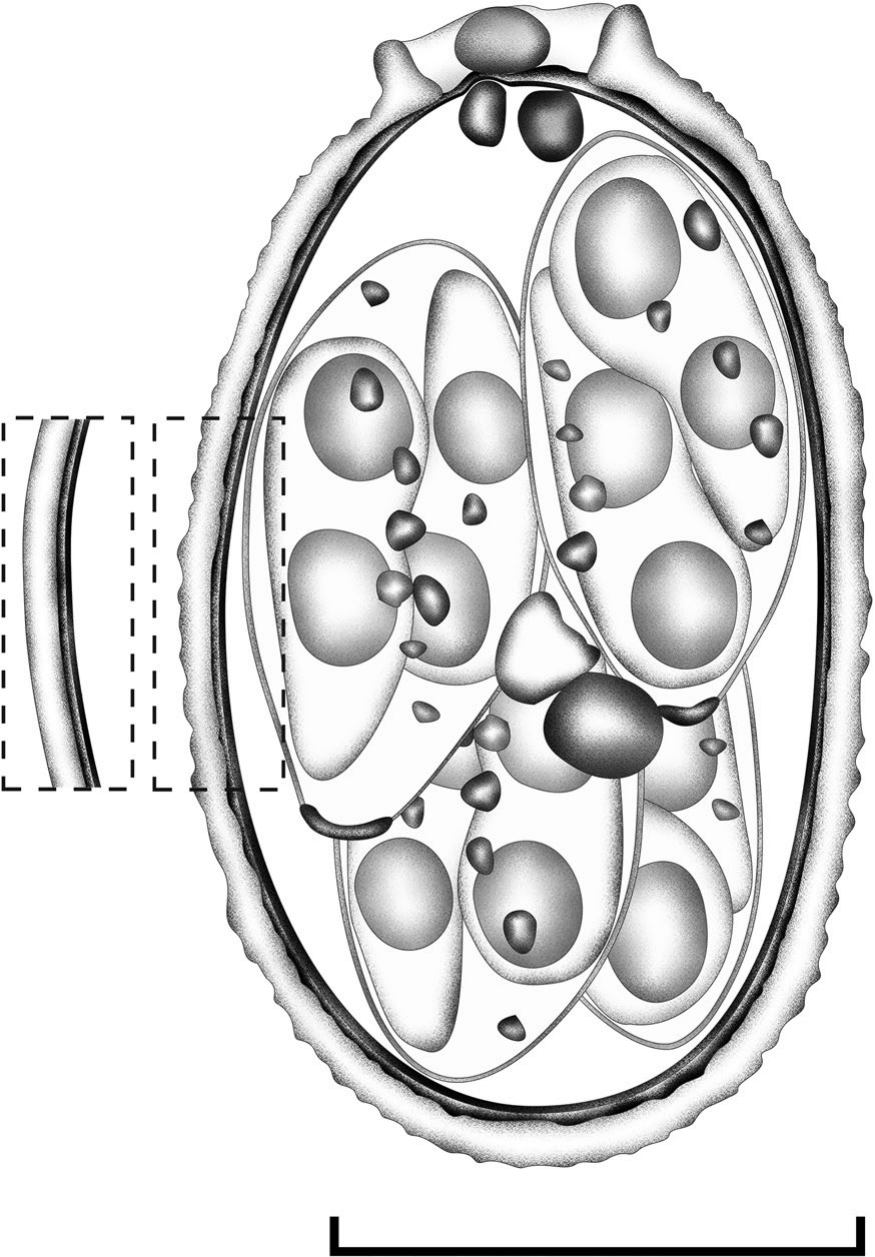
Composite line drawing of *E. haematopusi* **n. sp***.* sporulated oocyst. Scale bar = 20 µm

**Fig. 2:**
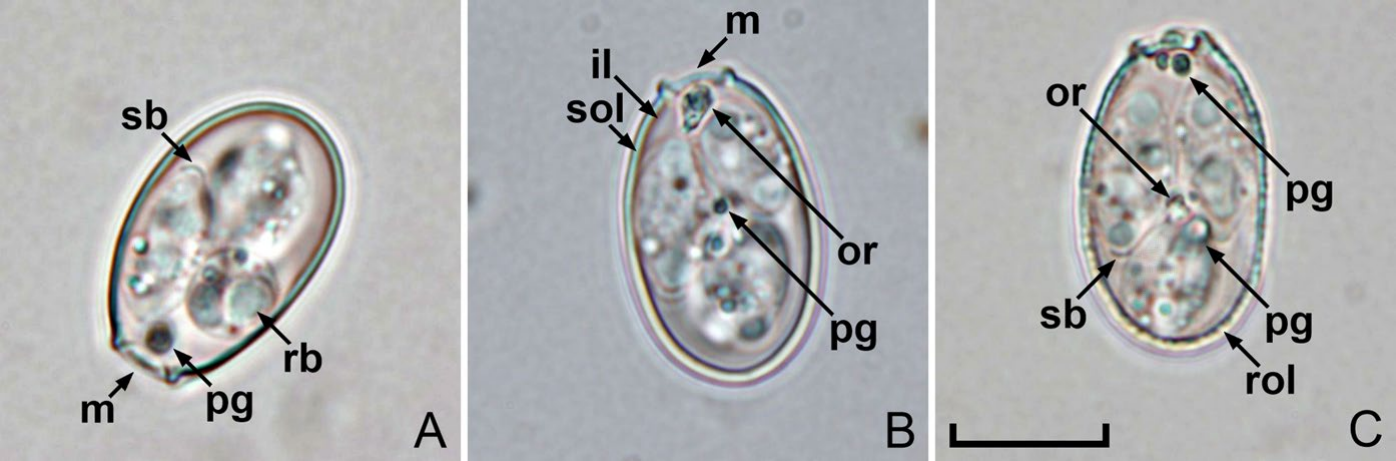
Photomicrographs of sporulated oocysts of *E. haematopusi* **n. sp**. from the Australian pied oystercatcher Haematopus longirostris. Note the inner layer (il) and smooth/rough outer layer (sol/rol) in the oocyst wall, micropyle (m), oocyst residuum (or), polar granule (pg), refractile body (rb) and Stieda body (sb). Scale bar: 10 μm

Oocysts (*n* = 20) ellipsoidal, 20–21 × 12–13 (20.1 × 12.9) μm; length/width (L/W) ratio 1.5–1.6 (1.56). Wall bi-layered, 0.7–1.2 (0.9) μm thick, outer layer smooth to slightly rough, c.2/3 of total thickness (Table 1.). Micropyle present, robust, and protruding, 3.5–4.3 (3.9) μm wide; occasionally with a rounded polar body attached to the micropyle. Oocyst residuum presents as a compact irregular mass, in addition to two to five polar granules being present. Sporocysts (*n* = 20) ellipsoidal, 9–11 × 5–6 (10.1 × 5.9) μm; L/W ratio 1.6–2.1 (1.71). Stieda body present, flattened to half-moon-shaped, 0.3–0.4 × 1.2–1.3 (0.4 × 1.3) μm; sub-Stieda body and para-Stieda body absent; sporocyst residuum present, composed of a few spherules scattered among the sporozoites. Sporozoites with anterior and posterior refractile bodies, but the nucleus is indiscernible (Fig. 1 & 2).

**Table 1:** Comparative morphology of *E. haematopusi* **n. sp** and Eimeria spp. recorded from wetland and grassland birds

Coccidia	Hosts	References	Oocysts					Sporocysts				
			Shape	Measurements (μm)	Shape index	Wall (μm)	Polar granule	Shape	Measurements	Stieda body	Sub-stieda body	Residuum
*E. bosquei*	Sandhill Cranes (*G. canadensis*)	Parker & Duszynski, 1986	Subspherical toovoid	23.6 × 17.1 (19.0–27.0 × 14.0–19.0)	1.38	Bi-layered c. 1.5	Present	Ovoid	12.3 × 9.3 (10.0–14 × 7–11)	Present	Present	Small, compact
*E. haematopi*	Eurasian oystercatcher (*H. ostralegus*)	Gottschalk and Prange, 2011	Ovoid	21.0 × 15.6 (18.0–25.2 × 14.4–18.2)	1.35	NA	NA	NA	NA	NA	NA	NA
*E. haematopusae* n. sp.	Australian pied oystercatcher (*H. longirostris*)	This study	Ellipsoid	20.1 × 12.9 (20–21 × 12–13)	1.56	Bi-layered c. 0.9	Present	Ellipsoidal	10.1 × 5.9 (9.0 -11.0 × 5.0–6.0)	Flattened	Absent	Composed of few spherules
*E. reichenowi*	Sandhill crane (*G. canadensis*)	Courtney et al., 1975	Ovoid-ellipsoid	17.8 × 15.3 (13–22 × 11–19)	1.32	Bi -layered c.0.4	Present	Ovoid	11.9 × 6.6 (9.0–14.0 × 5.0–8.0)	Present	Present	Composed of very fine faint granules
*E. gruis*	Sarus crane (*G. antigone*)	Courtney et al., 1975	Ellipsoid	18.0 × 11.4 (15–21 × 10–13)	1.58	Bi -layered c.1.25	Present	Ovoid	9.8 × 5.4 (9.0–11.0 × 5.0–7.0)	Present	Present	Composed of few spherules
*E. paludosa*	American coot (*F. americana*)	McAllister and Upton, 1990	Ovoid	16.5 × 12.6 (25–32 × 23–30)	1.31	One-layered c. 0.8	Present	Elongate-ovoid	10.8 × 6.2 (10–12 × 5–7)	0.6 × 1.5	1.2 × 2.0	Composed of very fine faint granules
*E. crecis*	Corn crake (*C. crex*)	Jeanes et al., 2013	Spherical	15.3 × 14.3 (13–18 × 12–16)	1.07	Bi-layered c.1.0	Present	Ovoid	9.2 × 5.4 (8–10 × 5–7)	Present	Present	Small, compact
*E. nenei*	Corn crake (*C. crex*)	Jeanes et al., 2013	Ellipsoidal	23.6 × 18.1 (21–26.0 × 17–20)	1.30	Bi-layered c. 0.8	Present	Ellipsoidal	12.6 × 6.5 (11–14 × 6–8)	Present	Present	Large, central, compact
*E. porphyrulae*	Purple gallinule (*P. martinica*)	Lainson, 1994	Ellipsoidal	22.4 × 17.7 (20–23.7 × 16.2 × 18.7)	1.30	Bi-layered	Present	Ellipsoidal	17.5 × 9 (17–19 × 8–10)	Present	Present	Scattered granules or compact mass
*E. gallinulae*	Dusky moorhen (*G. tenebrosa*)	Yang et al., 2014	Ovoid	17.3 × 13.3 (16.3–17.9 × 12.7–13.9)	1.30	Bi-layered c. 1.0	Present	Elongate-ovoid	8.4 × 5.1 (8.0–8.9 × 4.9–5.5)	Dome	Rectangular-shaped	Compact
*E. vanelli*	Yellow-wattled lapwing (*V. malabaricus*)	Mandal, 1965	Oval or pyriform	20.9 × 14.3	1.46	Na	NA	Pyriform	12.1 × 6.6	NA	NA	Present

Type-host: Australian pied oystercatcher (*Haematopus longirostris* Vieillot) (Aves: Charadriiformes: Haematopodidae)

Other hosts: Unknown.

Type-locality: Heirisson Island (− 31° 57′ 35.39″ S, 115° 52′ 34.19″ E), Perth, Western Australia, Australia.

Type-material: Oocysts in 10% formalin and oocyst photo-syntypes were deposited in the Western Australian Museum under the reference number WAM Z68805.

Prevalence: 1/1 (100%).

Prepatent period: Unknown.

Patent period: Unknown.

Site of infection: Unknown, oocysts collected from faeces.

Sporulation time: 48–72 hours.

Oocysts in 10% formalin and oocyst photosyntypes were deposited in the Western Australian Museum under the reference number WAM Z68803.

Representative DNA sequences: The newly generated sequences are deposited in the GenBank database under the accession numbers OR568560 and OR568561 for 18S rRNA Clone 1 and Clone 2, respectively.

ZooBank registration: To comply with the regulations set out in Article 8.5 of the amended 2012 version of the International Code of Zoological Nomenclature (ICZN, 2012) details of the new species have been submitted to ZooBank. The Life Science Identifier LSIDurn:lsid:zoobank.org:pub:2366D36B-A3C1-49A0-A330-FE958E0448B6. The LSID for the new name *Eimeria haematopusi* is LSIDurn:lsid:zoobank.org:act:5960EF64-64E7-4DA0-94B5-934B8801CC37.

***Phylogenetic analysis of 18S rRNA gene***

**Fig. 3:**
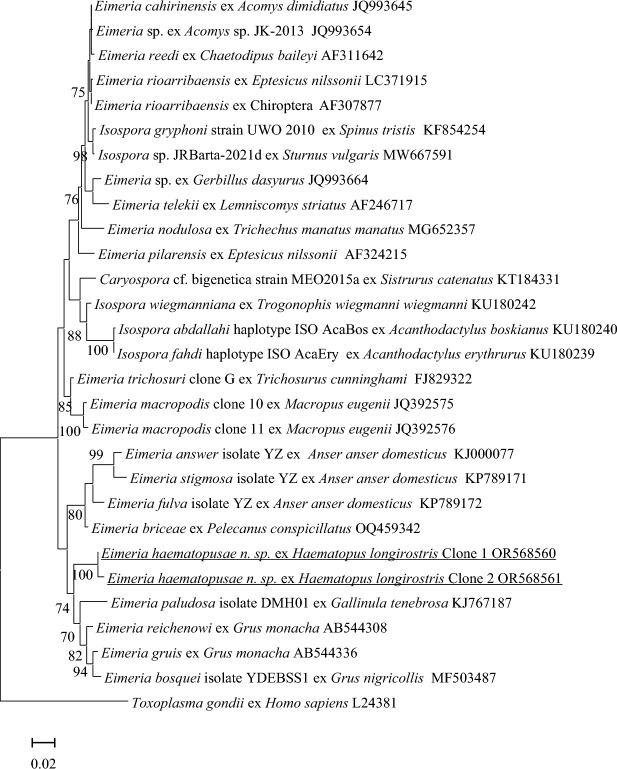
Evolutionary relationships of *E. haematopusi* n. sp. inferred by distance analysis of 18S rRNA sequences. Percentage support (>70%) from 1000 pseudoreplicates from ML analysis

A total of eight positive colonies containing the pGEMT- *E. haematopusi*18S rRNA vector were picked up and sequenced in both directions. Two types of unique sequences were obtained and were named Clone1 and Clone 2, at 1286 and 1289 bp, respectively. The two types of 18S rRNA sequences were aligned with 19 *Eimeria* spp., five *Isospora* spp. and one *Caryospora* sp. sequences isolated from birds. The justification for the selection of the reference sequences chosen was based on the NCBI Blast similarities (one sequence per species) and included sequences from *Eimeria* spp. relevant to this study. A sequence of *Toxoplasma gondii* (Nicolle and Manceaux) (EF472967) was used as the outgroup. The *E. haematopusi* **n. sp**. sequences from Clone 1 and 2 shared the highest genetic similarity of 97.6% and 97.2%, respectively with *E. reichenowi* (AB544308), which was identified from a hooded crane (*Grus monacha* Temminck) in Japan. As shown in Fig. 3, *E. haematopusi* **n. sp.** Clone 1 and 2 grouped within a clade with *Eimeria* spp. isolated from wetland birds and include *E. paludosa* (KJ767187) from a dusky moorhen (*Gallinula tenebrosa*) in Western Australia, *E. reichenowi* (AB544308) and *E. gruis* (AB544336) both from hooded cranes (*G. monacha*) and *Eimeria bosquei* (MF503487) from a black-necked crane (*Grus nigricollis* Przhevalsky) in China*.*

## Discussion

To our knowledge, there have been no previous reports of *Eimeria* species identified from the Australian pied oystercatcher (*H. longirostris*). This is the first description of *Eimeria* identified from an Australian oystercatcher.

Coccidia infections in birds may result in disease or may be subclinical. There are more than 197 *Eimeria* species infecting birds worldwide (Yabsley, 2008). Most of these complete their lifecycle in the alimentary tract. Enteric disease caused by pathogenic *Eimeria* species are responsible for large losses in the poultry industry worldwide. A small number of *Eimeria* species undergo extra intestinal development stages in the kidneys, for example *Eimeria truncata* in geese or throughout the body (disseminated visceral coccidiosis) as occurs with *Eimeria reichenowi* and *Eimeria gruis* in cranes (Samour, 2016). Both *Eimeria* and *Isopora* can commonly be found in the kidneys of wild geese, ducks, and shorebirds while hepatic coccidiosis due to *Eimeria* is seen in mutton birds and little penguins (Obendorf and McColl, 1980). In a previous study, *E. haematopi* was described from the kidneys of a Eurasian oystercatcher (*H. ostralegus*) during investigations of parasites in sea and coastal birds along the German North Sea coast (Gottschalk and Prange, 2011). In a similar study, coccidian infections were identified in two Eurasian oystercatchers (*H. ostralegus*) during histological examination of kidneys, during investigations of the health status of seabirds along the North Sea coast of Germany (Siebert et al., 2012). In the later study, the coccidia were not identified, limiting comparisons between *Eimeria* species isolated from *H. ostralegus*. Morphological and molecular characterization of the oocysts isolated from the pied oystercatcher in this study revealed that it was a novel species of *Eimeria.*

It would have been advantageous if histopathological studies had been performed on the bird in this study however the bird was sent for incineration before these studies could be conducted, It is also not possible to know if the coccidian infection played a role in the death of the bird as no necropsy was done and it may well have been an incidental finding.

Morphological comparison between the *Eimeria* species from the Australian and Eurasian oystercatchers showed oocysts of ***E. haematopusi*** **n. sp.** to be smaller in size and ellipsoid in shape compared to the oocysts of *E. haematopi* which are ovoid and larger. Unfortunately, no other morphological comparisons could be made between these two *Eimeria* species due to the limited features described for *E. haematopi* (Gottschalk and Prange, 2011) (http://eimeria.unl.edu/charad.html) (accessed on 20 Sep. 2023). When the morphological parameters of ***E. haematopusi*** **n. sp**. was compared to genetically similar *Eimeria* species *E. reichenowi*, *E. paludosa* and *E. gruis* no obvious similarities were identified. The oocysts of *E. reichenowi*, *E. paludosa* and *E. gruis* all measure smaller than *E. haematopusi* **n. sp**., have generally ovoid shaped sporocysts and present with a sub-Stieda body residuum. This is in contrast with *E. haematopusi* **n. sp**., which is larger in size, ellipsoidal in shape and lacks a sub-Stieda body residuum.

Unfortunately, no genetic comparisons could be made between the two *Eimeria* species (*E. haematopusi* **n. sp.** and *E. haematopi***)** isolated from oystercatchers to date, given the lack of genetic data for* E. haematopi*. Comparison between other *Eimeria* species however showed *E. haematopusi* **n. sp**. to be most closely related to *E. reichenowi *(AB544308), (genetic similarity of 97.6% and 97.2% from Clone 1 and 2, respectively) identified from a hooded crane (*G. monacha*) in Japan and less like *Eimeria briceae* from an Australian pelican (*Pelecanus conspicillatus* Temminck) with genetic similarities of 96.1% to Clone 1 and 95.6% to Clone 2. The close association of *E. haematopusi* **n. sp**. to *Eimeria* species isolated from a variety of different host species including the hooded crane (*G. monacha*) and dusky moorhen (*G. tenebrosa*) suggest that the evolution of this parasite does not solely occur along with their hosts but that the different ecological environments are also important in this long evolutionary process. Genetic comparisons between *Eimeria* isolates from different species of oystercatchers would be vital for such clarification.

In this study, we describe a novel species of *Eimeria* from the Australian pied oystercatcher and contribute to the first genetic characterisation of *Eimeria* species described from the Haematopodidae family. To understand the health impacts of this parasite, further studies are needed to ascertain the effects this *Eimeria* species has on its host.

## Conclusion

Morphological and genetic comparison of *E. haematopusi ***n. sp.** with other known *Eimeria* spp. revealed that the coccidian identified in the wild Australian pied oystercatcher is a new species. *E. haematopusi*** n. sp.** is the first coccidian to be reported from an Australian pied oystercatcher and is the first sequence available of a coccidian from this bird species. The morphological and genetic sequence information from our study of *E. haematopusi*** n. sp.** further contributes towards our knowledge of *Eimeria* spp.

## Data Availability

No datasets were generated or analysed during the current study.
